# fMRI of Retina-Originated Phosphenes Experienced by Patients with Leber Congenital Amaurosis

**DOI:** 10.1371/journal.pone.0086068

**Published:** 2014-01-21

**Authors:** Manzar Ashtari, Laura Cyckowski, Alborz Yazdi, Amanda Viands, Kathleen Marshall, István Bókkon, Albert Maguire, Jean Bennett

**Affiliations:** 1 Department of Radiology, Children's Hospital of Philadelphia, Philadelphia, Pennsylvania, United States of America; 2 Center for Cellular and Molecular Therapeutics, Children's Hospital of Philadelphia, Philadelphia, Pennsylvania, United States of America; 3 Vision Research Institute, Lowell, Massachusetts, United States of America; 4 F. M. Kirby Center for Molecular Ophthalmology, Scheie Eye Institute, University of Pennsylvania, Pennsylvania, United States of America; 5 Ophthalmology Department, Children's Hospital of Philadelphia, Philadelphia, Pennsylvania, United States of America; Dalhousie University, Canada

## Abstract

A phenomenon characterized by the experience of seeing light without any light actually entering the eye is called phosphenes or photopsias. Phosphenes can occur spontaneously or via induction by external stimuli. Previous reports regarding phosphenes have primarily focused on externally induced phosphenes such as by applying alternating or direct current to the cortex. A few of these reports used functional magnetic resonance (fMRI) to study activations induced by cortical phosphenes. However, there are no fMRI reports on spontaneous phosphenes originating from the retina and the resulting pattern of cortical activations. We performed fMRI during a reversing checkerboard paradigm in three LCA patients who underwent unilateral gene therapy and reported experiencing frequent phosphene on a daily basis. We observed bilateral cortical activation covering the entire visual cortices when patients reported experiencing phosphenes. In contrast, in the absence of phosphenes, activation was regulated by patient's visual ability and demonstrated improved cortical activation due to gene therapy. These fMRI results illustrate the potential impact of phosphene perception on visual function and they may explain some of the variability that clinicians find in visual function testing in retinal degeneration. Although we did not perform correlations between visual function and phosphenes, we hope data presented here raises awareness of this phenomenon and its potential effect on visual function and the implications for clinical testing. We recommend a thorough history for phosphene experiences be taken in patients with retinal disease who are candidates for gene or molecular therapy. Lastly, these data illustrate the potential power of fMRI as an outcome measure of gene therapy and the negative impact phosphenes may have on vision testing. fMRI has proven to be a sensitive, non-invasive, and reproducible test paradigm for these purposes and can complement standard visual function testing.

## Introduction

A phenomenon characterized by the experience of seeing light without any light actually entering the retina is called a phosphene. This phenomenon is also known as photopsias and the two terms have been used interchangeably in the literature. The most familiar type of phosphene is the pressure phosphene, which is elicited by pressing on the orbit, e.g. rubbing of the eyes. According to a pupil of Aristotle, it was Alcmaeon of Croton (circa 530–470 BC) who first reported that “the eye obviously has fire within, for when it is stuck this fire flashes out,” [1]. Phosphenes can occur not only through external stimuli, but also through drugs [2], [3], ionizing radiation [4]–[7], electrical [8]–[13], or magnetic stimulation [14]–[19] of the visual cortex, such as via Transcranial Magnetic Stimulation (TMS) [14]–[19]. Meister et al. [20] performed single-pulse TMS on 22 healthy subjects and observed that, with the same intensity TMS, some subjects experienced phosphenes and some didn't. The authors followed up their experiments with fMRI using checkerboard stimuli to investigate neurological differences between phosphene perceivers and non-perceivers. Subjects who did not report phosphenes had slightly greater activation in the primary visual cortex (V1). Subjects who did report phosphenes had greater extra striatal activation such as in V2/V3 areas. The authors concluded that the level of extra striatal activation seemed to be related to phosphene perception whereas the level of striate activation did not play a critical role, suggesting that phosphene perception may be related to the processing of information in V2 and V3 areas rather than V1. A more recent study [21] used simultaneous TMS and fMRI on 12 healthy subjects to record cortical activation induced by TMS. [21]. While all subjects demonstrated widespread cortical activations in response to TMS, subjects who reported seeing phosphenes had more significant and greater areas of activation within their visual cortex including the primary visual cortex [21]. A recent study using simultaneous TMS and electroencephalography (EEG) also concluded that phosphene perception arises after extensive processing in the brain. [22].

In addition to using magnetic stimulation, which induces electrical currents in the brain via changing magnetic fields, direct electrical stimulation of the occipital lobe also elicits phosphenes. Non-invasive electrical stimulation of the occipital lobe, such as transcranial Direct Current Stimulation (tDCS) of the V1 area has also been shown to elicit phosphenes [11], [12]. Furthermore, the non-invasive electrical stimulation technique, transcranial alternating current stimulation (tACS), over the occipital lobe has been shown to induce phosphenes as well [23]. In fact, using tACS has brought up the issue of whether the phosphenes are solely originating from the occipital area or whether there may be a component with retinal origin as well [13]. Schutter and colleagues [13] performed frontal and occipital tACS on 8 healthy volunteers and reported that tACS of the frontal lobe not only evoked phosphenes but that the phosphenes evoked by the frontal stimulation, reported by the same subjects, were significantly more intense than those evoked by occipital stimulation. Schutter et al [13] hypothesized that in the case of frontal lobe stimulation much of the current may have leaked through the scalp into the retina, and the reported phosphenes may have been caused by stimulation of the retina.

Beyond externally induced phosphenes, phosphenes may also occur spontaneously in individuals with diseased retinas, in normal subjects exposed to high-energy radiation [4]–[7] or taking certain medications [2], [3], or during intraocular insults, such as posterior vitreous detachment [24], [25] or in individuals with a range of retinal or CNS diseases, including retinal detachment or inflammatory, ischemic, autoimmune or degenerative disease [26], [27]. An example of fMRI from spontaneously generated phosphenes also presented in a case study of an individual with brain infarct [28]. In retinal degenerative diseases, phosphenes may be the manifestations of spontaneous activity in degenerating retinal cells due to remodeling of the inner retina or to an unregulated overproduction of free radicals that produce bio/chemiluminescent photons [29]. Chemiluminescent photons are then absorbed by the photoreceptors and initiate a photo-transduction cascade, which results in the perception of phosphenes [30]. Narici et al. [31] later revealed that the lipid peroxidation of the photoreceptors can produce (bio)chemiluminescent photons that generate anomalous visual effects, such as those associated with retinal phosphenes [31]. One of the most severe group of retinal degenerative diseases is Leber's Congenital Amaurosis (LCA), a disease characterized by profound impairment of the retina and poor visual function in infancy and early childhood followed by progressive degeneration and loss of retinal cells [32]–[34]. In our studies developing fMRI as an outcome measure for gene therapy intervention in LCA2, we were struck by the fact that many of the patients reported experiencing phosphenes, and a few on a daily basis, which was not revealed in their clinical history. Furthermore, there are no studies investigating fMRI results of patients with retinal degenerative disease reporting phosphenes. In this report we describe, for the first time, fMRI results from several LCA2 patients during episodes of phosphenes and in the absence of any phosphenes. We show that patients who spontaneously see phosphenes have immense and significant activation in their occipital lobes covering all retinotopic visual centers in the brain whereas they show minimal activation (depending on their age and stage of LCA) when they do not experience phosphenes. The results of this study demonstrate the impact that phosphenes have on vision and on fMRI testing in patients with LCA2 and suggest that queries into phosphene experience could be helpful for understanding test-to-test variations in vision testing.

## Materials and Methods

### A. Study Participants

Patients were selected from an ongoing neuroimaging study of LCA2 patients who had received unilateral subretinal injection of AAV2.hRPE65v2 [32], [33], [35] in a separate clinical study (www.clinicaltrials.gov NCT00516477; [32], [35]). fMRI results were previously reported for the first three patients who underwent fMRI after unilateral gene therapy (subjects CH08, CH09, and CH13) [34]. The present study aimed to characterize the effects of phosphenes on the fMRI-measured cortical response to reversing checkerboard stimuli, in three LCA2 patients (NP02, CH08, and CH11), from the eye treated with gene therapy (the right eye) and the left (untreated) eye. For comparison purposes we also present the fMRI results of two normally sighted controls that report not experiencing phosphenes (Table 1).

**Table 1:** Subject demographics.

Subject ID	Age at MRI (Years)	Sex	Left Eye Visual acuity (LogMAR)	Right Eye Visual acuity (LogMAR)	Experiences Phosphenes	Treated Eye	AAV2-hRPE65v2 dose (vg)/vol. (ml)	RPE65 Mutation	Time between AAV2 Inj. & “No Phosphene” MRI	Time between AAV2 Inj. & “Phosphene” MRI
CH06	20	F	1.74	1.34	Yes/Seldom	Right	4.8 E10/150	IVS1+5 g>a/L341S		
**CH08**	12	M	1.7	0.76	**Yes/Frequent**	Right	4.8 E10/150	F530fs/F530fs	3 Yrs	3.5 Yrs
CH09	8	M	.76	1.03	No	Left	4.8 E10/150	R124X/Lys297del1aggA		
CH10	10	M	0.88	0.69	Yes/Seldom	Right	4.8 E10/150	IVS1+5 g>a/Phe530del1ttc		
**CH11**	26	F	0.76	0.64	**Yes/Frequent**	Right	4.8 E10/150	V473D/V473D	2 Yrs	2 Yrs
CH12	44	F	3.25	3.25	No	Right	1.5 E11/300	K303X/W431C		
CH13	35	M	1.61	1.53	No	Right	1.5 E11/300	IVS1+5g>a/IVS1+5g>a		
NP01	30	F	1.60	1.77	No	Right	1.5 E10/150	E102K/ E102K		
**NP02**	30	M	1.71	0.96	**Yes/Frequent**	Right	1.5 E10/150	E102K/ E102K	4Yrs	4Yrs
NP15	11	M	0.58	0.48	Yes/Seldom	Left	1.5 E11/300	D167W/H313R		
**Controls**										
NC04	30	M	0	0	NA	N/A	NA	NA	NA	NA
NC05	25	F	0	0	NA	N/A	NA	NA	NA	NA

### B. Ethics Statement

The institutional review board (IRB) at the Children's Hospital of Philadelphia (CHOP) under the IRB protocol number 09-007165 approved this study. After a complete description of the fMRI study was provided, written informed consent (and when necessary, parental permission consent and child assent) was obtained from all subjects. The CHOP Institutional Review Board (IRB) approved all study procedures. All patients had been clinically assessed as part of their qualification to enter the clinical trial for retinal gene therapy [32], [33], [35].

### C. Magnetic Resonance Imaging

#### C1. Image Acquisition

MRI scans were conducted at The Children's Hospital of Philadelphia (CHOP) on a 3T Siemens Verio system dedicated to research using a 32 channel head coil. All scans were carried out by a single operator and monitored to be free of artifacts at the time of acquisition.

#### *C1.1. Functional MR (fMRI)*

Functional data were acquired using the blood oxygenation level dependent (BOLD) technique acquiring 90 functional volumes of 46 slices with 3 mm isotropic resolution (Matrix = 64×64; TR/TE 3000/30 ms) with a total acquisition time of 4∶39 minutes. To permit T1 saturation, three additional volumes were acquired at the beginning of the fMRI experiment, which were not used in image analysis. A transistor-transistor logic (TTL) pulse was used to automatically start the stimuli in-synch with the start of fMRI acquisition. An MRI compatible response device (a button that the subject pushed when recognizing the stimulus) was used to record subject responses. Subjects were instructed to press the button once when the checkerboard first appeared and, if they experienced phosphenes, to press and hold the button for the duration of the phosphene. If the subjects reported seeing phosphenes, additional acquisitions were performed until a phosphene free session was acquired. However, in the present study we analyzed the acquisitions in which subjects reported phosphenes and compared them to acquisitions in which no phosphenes were reported for that subject.

#### *C1.2. Real Time Patient Monitoring*

**Figure 1:**
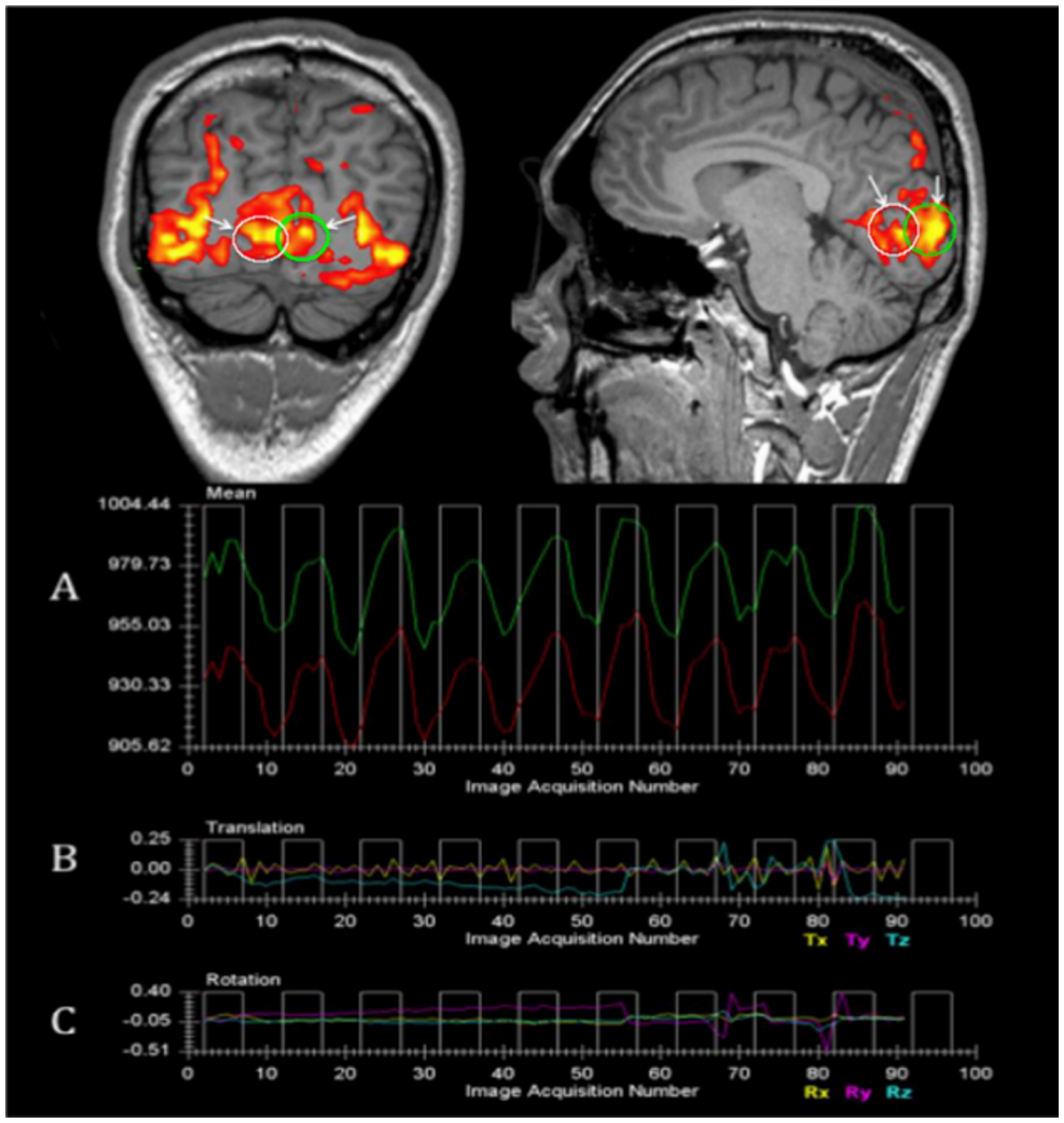
Real time patient monitoring. Real time fMRI software allows continuous monitoring of subject's cortical response for specific regions of interest (ROIs) placed in the area of interest. In the example above, two ROIs (white and green circles shown with white arrows) are selected in the right and left primary visual cortex (V1) located in the medial aspect of the occipital lobes encompassing the calcarine fissures. Real time fMRI monitoring also allows motion detection in three translational and three rotational orientations depicted in the B and C panels respectively. Experiments are interrupted if no response is detected in panel A or the rotational and translational movements are>0.6.

Real time monitoring of patient motion and task performance was performed using the real time fMRI feature available on the Verio research MR system (Figure 1). As shown in the coronal and sagittal views located at the top section of Figure 1, two regions of interest (ROI) within the right and left primary visual cortex (V1) are selected (green and white circles depicted by white arrows) that are located in the medial aspect of the occipital lobes encompassing the calcarine fissures to monitor patients performance. The bottom panel shows how the patient is being monitored in real time. Experiments are interrupted if no response is detected in panel A and/or the rotational and translational movements depicted in panels B and C are >0.6. This feature allows reliable acquisition of fMRI from each participant and confirms that the subject is alert and awake and performing the task. In cases where the subject may have fallen asleep or may not have been paying attention to the task, the green and red lines would disappear in panel A.

#### *C1.3. 3D T1 Weighted (MPRAGE)*

A 3D isotropic structural high resolution T1 sequence with inversion preparation pulse was obtained for visual activation localization and generation of inflated and flat hemispheres (IR-Prep: T_R_ = 2080 ms, T_E_ = 2.54 ms, BW = 180 Hz/Px, matrix size = 320×320, FOV = 256×256 mm^2^, 192 axial slices, slice thickness = 0.8 mm, inversion time = 1200 ms with Flip Angle = 8°, NEX = 1, Echo Spacing = 7.8 iPAT = 2 and scan time = 7∶04 minutes).

#### C2. fMRI Visual Paradigm

**Figure 2:**
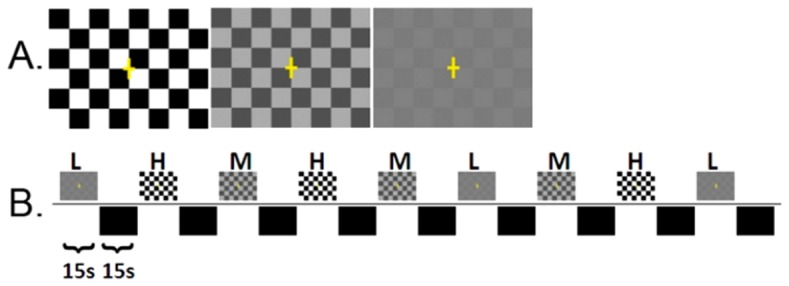
fMRI stimuli and paradigm design. (A) Checkerboard stimuli with a constant light intensity of 5 lux at three levels of contrast: high (H; 100%), medium (M; 34%) and low (L; 10%), were presented in a boxcar block design. (B) The checkerboard paradigm consisted of 15 sec active blocks of contrast reversing (8 Hz) checkerboards interleaved with 15 sec presentations of a blank (black) screen as control blocks (rest period). Three blocks of each contrast were interspersed randomly and interleaved with nine rest blocks.

Checkerboard patterns with a constant (low) light intensity of 5 lux were used in a block design fMRI paradigm with 3 levels of contrast: high, 100%; medium, 34%; low, 10% [34]. As shown in Figure 2 the checkerboard paradigm consisted of 15-second active blocks of contrast-reversing (8-Hz) black and white checkerboards interleaved with 15 seconds of blank (black) screens as control blocks. Subjects were asked to fixate on the yellow cross in the center of the pattern, or, if they could not see the cross, were asked to look straight ahead. Stimuli were presented to one eye at a time and the switch between eyes took place electronically, thus each subject completed the task for each eye separately. Resonance Technology VisuaStim (Northridge, CA) goggles featuring a digital display of 500,000 pixels per 0.25 square inch, and a 30° horizontal field of view were used to present the fMRI stimuli unilaterally to each eye. The visual paradigm was programmed in E-Prime (Psychology Software Tools, Inc., Pittsburgh, PA).

#### C3. fMRI Image Preprocessing

All functional data from individual subjects were processed using BrainVoyager-QX (Brain Innovations, Maastricht, The Netherlands) [36]. Pre-processing of data included slice scan time correction, 3D motion correction, spatial smoothing, and temporal filtering. Since interpolation was used for scan time correction to insure that all voxels in the volume represented the signal simultaneously. A high-pass temporal filter of 2 cycles/run was applied to remove signal drift. Spatial smoothing was performed using a 3 mm full-width at half-maximum (FWHM) Gaussian filter. In addition to real time monitoring of subject motions, to rule out excessive motion, all functional data sets were additionally processed using the motion correction algorithm implemented in BrainVoyagerQX that calculates head translation (in millimeters) and rotation (in degree) for each volume in relation to the first volume. None of the subjects showed excessive motion during the scan (≥0.6 mm). Data was analyzed in BrainVoyagerQX using the general linear model (GLM). Each condition (high, medium, low contrast) was analyzed by specifying a design matrix defined as blocks with checkerboard presentation (active) versus blocks with blank black screen (rest) conditions followed by application of the hemodynamic response function and corrected for multiple comparisons using the false discovery rate (fdr). Although subjects reported seeing phosphene randomly during one of the high, low, or medium contrasts, for clarity fMRI results presented here are all from when subjects experienced phosphenes during the high contrast stimuli (high contrast – rest). However, it is important to mention that the intensity of cortical activations for the phosphenes occurring during the medium and low contrast stimuli were highly comparable to those reported here for the high contrast condition. fMRI results for the LCA patients are presented for the left and the right eyes with and without phosphenes to demonstrate the magnitude and distribution of cortical activations in the presence of phosphene in comparison to its absence. fMRI results for the presence of phosphene in all three LCA patients are presented at the most strict statistical threshold of fdr<3% along with a continuous connected area of >1000 mm^2^. In contrast, fMRI data in the absence of phosphene are presented at a much more relaxed statistical threshold.

## Results

### A. Demographics and Overall Phosphene Experiences

Demographic information, RPE mutation type, dose/volume of AAV2-hRPE65v2, and time between fMRI and gene therapy are presented in Table 1 [35]. Three of the ten clinical trial participants (CH08, NP02, and CH11; Table 1) reported that they had experienced phosphenes their entire lives, often several times in one day. These were characterized variously as moving horizontal and/or vertical lines, circles or semi-circular shapes, crescent moons, lightning bolts, swirling waves, and flashes covering the entire visual field. In addition to their transient photopsias, some patients reported experiencing constant background noise with sparklers or statics. The patients indicated that when it occurs they have to wait for it to be over before they can continue with their activities. There was no clear correlation of phosphene experience with other disease variables. For example, NP01 and NP02 are fraternal twins and are homozygous for the *RPE65* E102K mutation (Table 1), but only NP02 experiences phosphenes. Phosphenes can be experienced by young individuals (CH08, age 12) or by individuals in their late twenties, or early thirties (CH11, NP02; Table 1). As depicted in Table 1, all three LCA patients presented with varying measures for visual acuity and no correlation was observed between phosphenes and visual acuity.

### B. fMRI Results and Phosphene History of LCA2 Patients

Specific characteristics for significant clusters of brain activations such as cluster size, Talairach coordinates for the centroid cluster location, cluster statistics, and neurological identification of the activated regions along with designated Brodmann areas (BA) are presented in Tables 2 and 3 for LCA2 patients during non-phosphene and phosphene experiences, respectively.

**Table 2:** Cluster information of activated brain regions for LCA2 patients while not experiencing phosphenes.

Subject ID/Eye	Cluster Location (Centroid)	Cluster Size (mm^2^)	Centroid X	Centroid Y	Centroid Z	t-Value	p-Value
CH08/Left	Left lingual gyrus, BA 18	628	−10	−86	−4	3.9670	0.0002
	Left middle occipital gyrus, BA 18	548	−30	−94	3	4.2950	0.0000
	Right middle occipital gyrus, BA 18	625	27	−83	−8	4.0176	0.0001
	Right cuneus, BA 17	322	17	−83	9	3.5775	0.0006
	Right middle occipital gyrus, BA 18	407	27	−83	−8	3.9160	0.0002
	Right lingual gyrus BA 17	977	9	−96	−9	4.0441	0.0001
CH08/Right	Left middle occipital gyrus, BA 18	2041	−22	−89	5	6.1174	0.0000
	Left lingual gyrus, BA 18	2637	−24	−74	−7	7.3997	0.0000
	Left middle occipital gyrus, BA 19	2388	−35	−77	6	7.5364	0.0000
	Right fusiform gyrus, BA 19	2801	37	−74	−12	8.5140	0.0000
	Right cuneus, BA 18	5123	12	−85	21	7.2910	0.0000
	Right lingual gyrus, BA 18	4931	13	−76	6	7.5045	0.0000
CH11/Left	Left lingual gyrus, BA 18	350	−7	−92	−12	12.1390	0.0000
	Left cuneus, BA 17	222	−7	−101	−2	17.1579	0.0000
	Left lingual gyrus, BA 18	414	−10	−59	3	11.5825	0.0000
CH11/Right	Left lingual gyrus, BA 17	558	−7	−90	−6	3.7612	0.0003
	Left middle occipital gyrus, BA 18	1636	−19	−85	−8	7.1139	0.0000
	Left inferior occipital gyrus, BA 18	2426	−32	−92	−9	11.6948	0.0000
	Left inferior occipital gyrus BA 18	5432	−40	−83	−3	8.4111	0.0000
	Right lingual gyrus, BA 18	3908	17	−75	−9	6.0228	0.0000
	Right lingual gyrus, BA 17	242	10	−95	0	3.3365	0.0013
	Right lingual gyrus, BA 18	1025	5	−86	−1	6.0472	0.0000
	Right cuneus, BA 17	223	11	−74	11	3.1100	0.0025
NP02/Left	Left middle occipital gyrus, BA 19	6937	−31	−88	10	5.7497	0.0000
	Left fusiform gyrus, BA 19	1302	−31	−65	−7	5.3607	0.0000
	Left middle occipital gyrus, BA 19	1681	−26	−94	18	6.2505	0.0000
	Right lingual gyrus, BA 17	11962	21	−90	2	6.2937	0.0000
NP02/Right	Left middle occipital gyrus, BA 18	1483	−31	−90	3	4.2953	0.0000
	Right middle occipital gyrus, BA 18	2557	28	−92	2	4.1568	0.0001

**Table 3:** Cluster information of activated brain regions in LCA2 patients when experiencing phosphenes.

Subject ID/Eye	Cluster Location (Centroid)	Cluster Size (mm^2^)	Centroid X	Centroid Y	Centroid Z	t-Value	p-Value
CH08/Left	Left inferior occipital gyrus, BA 18	34431	−35	−96	−15	13.2272	0.0000
	Right cuneus, BA 17	29409	16	−80	14	9.7000	0.0000
CH08/Right	Left cuneus BA 17	46849	−17	−96	2	19.6190	0.0000
	Right lingual gyrus, BA 18	40249	15	−83	−3	15.0627	0.0000
CH11/Left	Left middle occipital gyrus, BA 18	22469	−28	−87	−3	13.3473	0.0000
	Right middle occipital gyrus, BA 18	24523	29	−83	3	15.2940	0.0000
CH11/Right	Left lingual gyrus, BA 18	25039	−6	−77	−3	13.8245	0.0000
	Right cuneus, BA 17	31109	5	−71	12	12.5168	0.0000
NP02/Left	Left lingual gyrus, BA 18	44082	−22	−74	−1	16.4914	0.0000
	Right middle occipital gyrus, BA 19	46505	32	−85	11	23.1436	0.0000
NP02/Right	Left middle occipital gyrus, BA 19	44352	−34	−79	3	12.3921	0.0000
	Right middle occipital gyrus, BA 19	44440	32	−85	11	12.3134	0.0000

The detailed information on phosphene experiences and fMRI results for LCA2 patients are as follows:

NP02, a 31 year old Caucasian male, reported seeing spontaneous flashes in his left eye almost every day, several times a day, but reported not experiencing phosphenes in his right eye as often. Notably, NP02 had received subretinal gene therapy four years prior to his MRI scan [32] (Table 1). He reported that the flashes of light were always composed of bright white glows (not colored) that generally lasted a few seconds. NP02 described his phosphenes as having the shape of a half moon (crescent shape) that move around and at times rotate in his field of vision and sometimes appear in the shape of vertical lines that move horizontally in his visual field. NP02 also reported seeing dimmer twinkling star-like flashes but less often. He also added that he sees phosphenes both when his eyes are open and closed, but he mostly experiences them in dark environments. He added that his phosphenes never occur in bright daylight. NP02 also reported that he is unable to see his surroundings while experiencing phosphenes.

**Figure 3:**
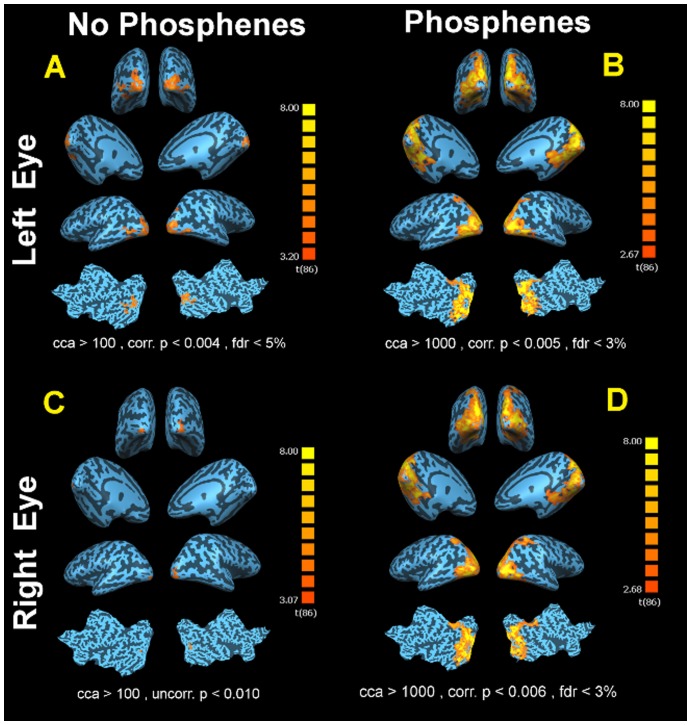
fMRI results for NP02 left and right eye (treated eye) with and without experiencing phosphenes. (A) fMRI results from the left eye when presented with the same checkerboard stimuli, when no phosphene was reported, showed significant clusters of activations (fdr<5%, Corrected p<0.004, cca ≥100 mm^2^) which were primarily distributed on the lateral aspects of visual cortex with minimal spread to the medial surface of the right hemisphere. (C) fMRI results from the right eye (treated eye) when presented with the same checkerboard stimuli while no phosphene reported by the subject (Uncorrected p<. 010, cca>100 mm^2^). (D) Similar to the left eye, visual cortex showed extraordinary amount of activations in response to the checkerboard stimuli presented to the right eye (treated eye) in the presence of phosphenes. fMRI results showed significant widespread (fdr<3%, Corrected p<0.006, cca ≥1000 mm^2^) bilateral activations extending and spreading to all visual centers of occipital cortex. (B) fMRI results for the left eye when presented with checkerboard stimuli showed significant widespread (fdr<3%, Corrected p<0.005, cca ≥1000 mm2) activations that were bilaterally distributed to all areas of visual cortex, when NP02 reported experiencing phosphene.

fMRI results from the left (untreated) and right (treated) eye of NP02 are presented in Figure 3. As depicted in panel A, in the absence of phosphenes, NP02 presented with significant activation for his untreated eye (left) (cca>100 mm^2^, Corr. p<0.004, fdr<5%) as compared to his treated eye (cca>100 mm^2^, Uncorr. p<0.010), shown in panel C. Notably, his treated retina had a macular hole [32], [33]. In the presence of phosphene, NP02 had sizeable significant (fdr<3%, Corr. p<0.006, cca ≥1000 mm^2^) and widespread bilateral cortical activations in response to the same checkerboard stimuli presented to his right (gene therapy-treated) eye (Panel D). Similar to his right eye, in the presence of phosphene, NP02 also showed similar significant widespread (fdr<3%, Corr. p<0.005, cca ≥1000 mm^2^) activations that also were bilaterally distributed to all areas of visual cortex when he was presented with the same checkerboard stimuli to his left eye (Panel B). In summary, there was not a clear improvement in cortical function in the gene therapy-treated eye, however phosphene perception in the untreated eye still resulted in strong activation of widespread cortical areas.

CH08, a 13 year old Caucasian male, reported spontaneous black and white flashes in both eyes. He also reported seeing moving horizontal or vertical lines and sometimes wave patterns of varying intensities. He reported seeing phosphenes both when his eyes are open and closed, but said he sees them more frequently in dark environments. CH08 reported that the frequency of his phosphenes has decreased somewhat since gene therapy (3 years prior to the fMRI) but still occurs often. CH08 also stated that the phosphenes last for a few seconds and he is not able to see when they occur.

**Figure 4:**
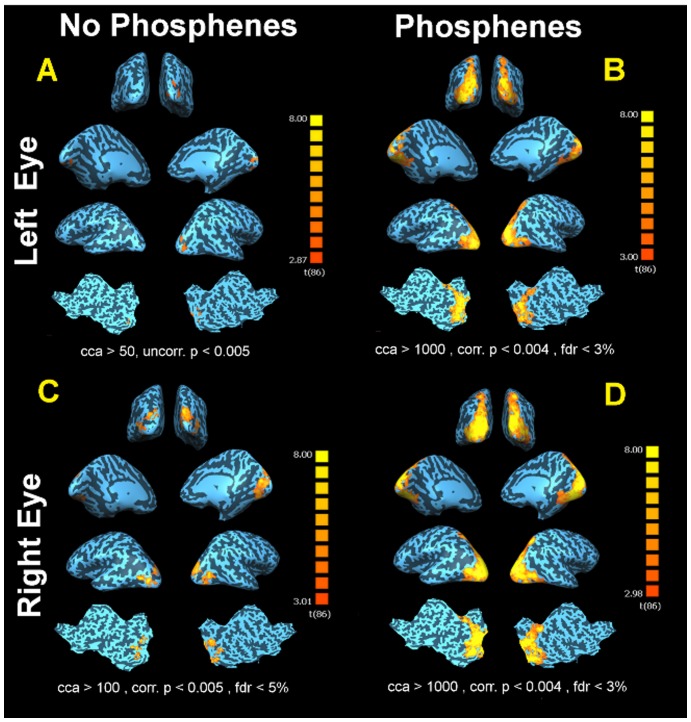
fMRI results for CH08 left and right eye (treated eye) with and without experiencing phosphenes. (A) fMRI results from the left eye when presented with the same checkerboard stimuli and no phosphene was reported showed small areas of activations detectable only at a less stringent statistical threshold (not fdr corrected) and much lower extent threshold (Uncorrected p<0.005, cca ≥50 mm^2^). (C) fMRI results from the right eye (treated eye) when presented with the same checkerboard stimuli and no phosphene reported by the subject. CH08 presented with significant activations for his treated eye (fdr<5%, Corrected p<0.005 cca>100 mm^2^) as compared to his untreated eye (left). However, although there are noticeably more activations for the treated eye, the magnitude and distribution of cortical activations for the treated eye when experiencing phosphene are significantly greater (D). (B) fMRI results showed significant widespread (fdr<3%, Corrected p<0.004, continuous connected area (cca) ≥1000 mm^2^) bilateral activations in all areas of visual cortex extending from medial to lateral and posterior to anterior aspects of the occipital cortex after presentation of checkerboard stimuli to his left eye while experiencing phosphene. (D) Similar to the left eye, visual cortex showed remarkable response to the checkerboard stimuli presented to the right eye (treated eye) while under the influence of phosphenes. fMRI results showed significant widespread (fdr<3%, Corrected p<0.004, cca ≥1000 mm^2^) bilateral activations extending and spreading to all brain visual centers.

fMRI results for CH08's left (control) and right (treated) eyes with and without experiencing phosphenes are presented in Figure 4. In the absence of phosphenes (Panel A, Figure 4) CH08 showed small areas of activations detectable only at a less stringent statistical threshold (not fdr corrected) and much lower extent threshold (uncorrected p<0.005, cca ≥50 mm^2^). In contrast, fMRI results from CH08's right (treated) retina in the absence of phosphenes (panel C) showed significant activation (fdr<5%, corrected p<0.005, cca>100 mm^2^), as compared to his untreated (left) eye.

When phosphenes were present (panel B), and stimuli were presented to the control eye, fMRI results showed significant widespread (fdr<3%, corrected p<0.004, continuous connected area (cca) ≥1000 mm^2^) bilateral activations in all areas of the visual cortex extending from medial to lateral and posterior to anterior aspects of the occipital cortex. A similar result was found when the right (treated) eye was stimulated with light while the subject perceiving phosphenes (panel D). There was significant widespread (fdr<3%, corrected p<0.004, cca ≥1000 mm^2^) of bilateral activations extending and spreading to all visual centers for the treated eye.

CH11, a 28 year old female, reported seeing two types of spontaneous black and white flashes in both her eyes. The first kind occurs almost all the time and is described as a black and white background noise similar to a TV screen with no reception. The second kind occurs less frequently but contains much brighter light intensity and is shaped like circles or lightning bolts. Similar to other subjects, CH11 reported seeing phosphenes with both open and closed eyes and her phosphenes occur more frequently in dark environments. CH11 reported that her phosphene experiences have decreased slightly since her gene therapy administration to the right eye, two years prior to the fMRI study. Also similar to other subjects, CH11's phosphene experiences last a few seconds during which her vision is obscured.

**Figure 5:**
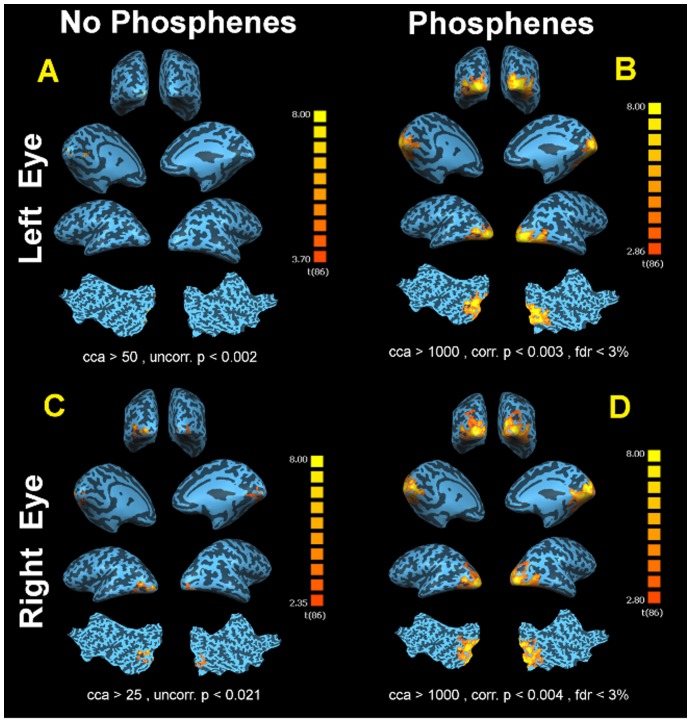
fMRI results for CH11 left and right (treated) eye with and without experiencing phosphenes. (A) fMRI results from the CH11 left eye, when presented with the same checkerboard stimuli and phosphenes were absent, showed limited areas of activations detectable only at a less stringent statistical threshold (Uncorrected p<0.010, cca ≥100 mm^2^). (C) fMRI results from the right eye (treated eye) when presented with the same checkerboard stimuli and no phosphene was reported. CH11 presented with significant activations for her treated eye (Uncorrected p<0.01, cca>100 mm^2^) as compared to her untreated eye (left). However, although there is noticeably more activation for CH11's treated eye, the magnitude and distribution of cortical activations for the treated eye when experiencing phosphene (D) are significantly more. (B) fMRI results showed significant widespread (fdr<3%, Corrected p<0.003, cca ≥1000 mm^2^) bilateral activations in all areas of visual cortex extending from medial to lateral and posterior to anterior aspects of the occipital cortex after presentation of checkerboard stimuli to the left eye while experiencing phosphene. (D) Similar to the left eye, visual cortex showed significant widespread responses (fdr<3%, Corrected p<0.004, cca ≥1000 mm^2^) to the checkerboard stimuli presented to the right eye (treated eye) while CH11 reported seeing phosphenes.

fMRI results for CH11's left and right eye (treated eye) with and without phosphenes when presented with reversing checkerboard stimuli are presented in Figure 5. As depicted in Figure 5, panel A, in the absence of phosphenes, results for CH11's left (untreated) eye during checkerboard stimuli showed limited areas of activations detectable only at a less stringent statistical threshold (Uncorrected p<0.002, cca ≥50 mm^2^). In contrast, when the right (treated) eye was stimulated in the absence of phosphenes (panel C), CH11 presented with larger activations (Uncorrected p<0.021, cca>25 mm^2^) compared to the untreated eye. When CH11 experienced phosphenes and the stimulus was presented to her untreated left retina, fMRI results showed significant widespread (fdr<3%, Corrected p<0.003, cca ≥1000 mm^2^) bilateral activations in all areas of the visual cortex extending from medial to lateral and posterior to anterior aspects of the occipital cortex (panel B). Similarly, the visual cortex showed significant widespread responses (fdr<3%, Corrected p<0.004, cca ≥1000 mm^2^) to the checkerboard stimuli presented to the right eye (treated eye) while CH11 reported phosphenes (panel D).

### C. fMRI Results of Sighted Controls

**Figure 6:**
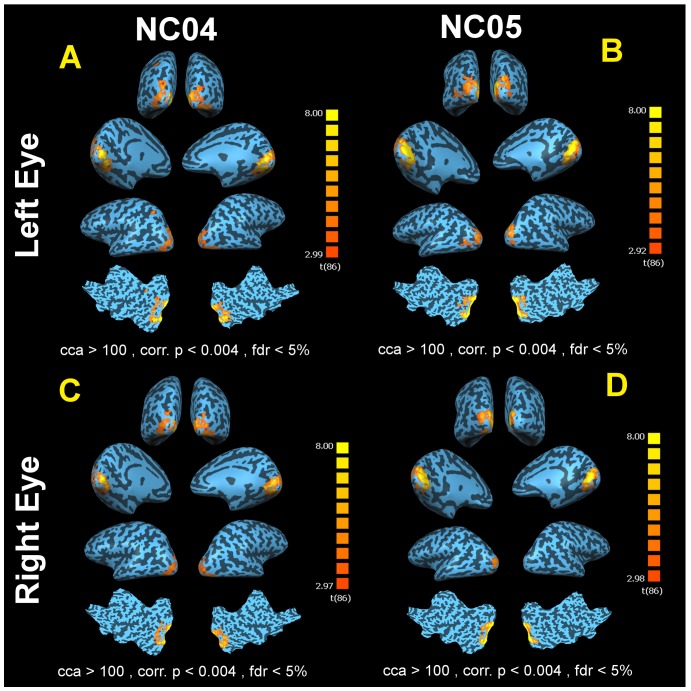
fMRI results for normal controls NC04 and NC05 left and right eye. (A) Left eye of NC04. (C) Right eye of NC04. (B) Left eye of NC05. (D) Right eye of NC05. Both subjects show a more normal activation pattern associated with the checkerboard stimuli (fdr<5%, Corrected p<0.004, cca>100 mm^2^). Note that the amount of activations seen in the normal control subjects is noticeably less than that of subjects experiencing phosphene. It is also important to point out that the activation pattern for both NC04 and NC05 in response to the checkerboard stimuli is more medially focused around the calcarine fissure than the more widespread pattern of activations seen in cases of phosphene. As shown here, the cortical activation pattern for the LCA patients, in the absence of phosphene, is drastically different from those of normal controls where activation is more focused in the primary visual cortex and symmetrically distributed between hemispheres for both the left and right eyes.

Table 4 summarizes the characteristics of significant clusters of activations for the two sighted controls depicting the size and Talairach coordinates for centroid location of each cluster, cluster statistics and specific Brodmann areas of activated brain areas. fMRI results for the left and right eye of two sighted controls using the same reversing checkerboard stimuli are presented in Figure 6. fMRI results for NC04's left and right eyes (fdr<5%, Corrected p<0.004, cca>100 mm^2^) are presented in panels A and C, respectively. fMRI results for NC05's left and right eyes (fdr<5%, Corrected p<0.004, cca>100 mm^2^) are presented in panels B and D, respectively. As depicted in Figure 6, the pattern of activation for both NC04 and NC05 in response to the checkerboard stimuli is more medially focused around the calcarine fissure compared to the more widespread pattern of activations seen in the individuals with LCA during phosphenes.

**Table 4:** Cluster information of activated brain areas for sighted controls.

Subject ID/Eye	Cluster Location (Centroid)	Cluster Size (mm^2^)	Centroid X	Centroid Y	Centroid Z	t-Value	p-Value
NC04/Right	Left middle occipital gyrus, BA 18	5937	−25	−86	−1	7.3937	0.0000
	Left middle occipital gyrus	552	−22	−86	7	5.6196	0.0000
	Right lingual gyrus, BA 17	24330	17	−86	4	10.5394	0.0000
	Left lingual gyrus, BA 18	14765	−16	−80	−6	10.4206	0.0000
NC04/Right	Left middle occipital gyrus, BA 19	795	−22	−87	8	4.7038	0.0000
	Right middle occipital gyrus, BA 18	20466	23	−85	−6	8.9940	0.0000
	Left cuneus, BA 18	22276	0	−93	10	19.7357	0.0000
NC05/Left	Left middle occipital gyrus, BA 19	966	−40	−68	3	5.9692	0.0000
	Right inferior occipital gyrus, BA 18	850	32	−84	−3	4.5530	0.0000
	Right middle occipital gyrus, BA 19	21298	36	−64	−1	19.4853	0.0000
	Left lingual gyrus, BA 18	16057	−4	−73	−1	12.0988	0.0000
NC05/Right	Left cuneus, BA 18	1926	−20	−95	13	5.8124	0.0000
	Right lingual gyrus, BA 18	13312	2	−74	−3	11.5988	0.0000
	Right cuneus, BA 18	825	12	−77	−14	5.0782	0.0000

## Discussion

A phenomenon characterized by the experience of seeing light without any light actually entering the eye is called phosphenes or photopsias. Phosphenes can occur spontaneously or via induction by external stimuli. Previous reports regarding phosphenes have primarily focused on externally induced phosphenes from the occipital cortex using magnetic stimulation[14]–[19], alternating [23] or direct current [11], [12]. Beyond externally induced phosphenes, phosphenes may also occur spontaneously [4]–[7], [23]–[26]. Only a hand full of these reports used fMRI to study activations induced by these phenomenon. A more recent study [21] used fMRI on 12 healthy subjects to test the direct relationship between the threshold of TMS and phosphene perceptions. Authors showed a threshold dependence for TMS induced phosphenes and reported significantly greater distribution of cortical activations for subjects who experienced the induced phosphenes as opposed to those who did not [21]. In addition to stimulated phosphenes, fMRI from spontaneous phosphene was presented from a case report on an individual with brain infarct [28].

In a separate self-report study Schutter and Hortensius [13] examined a group of normal controls undergoing frontal and occipital stimulations to compare the intensity of phosphenes created by the retina as compared to those originated from the occipital cortex. Based on the self-report of study participants, phosphenes evoked by the frontal stimulations, which are attributed to retinal origin, were significantly more intense than those evoked by occipital stimulations [13].

In retinal degenerative diseases, phosphenes may be the manifestations of spontaneous activity in degenerating retinal cells. In the current report we present fMRI results from 3/10 individuals with LCA2 who participated in an ongoing gene therapy clinical trial and neuroimaging study and reported experiencing phosphenes on a daily basis. The phosphene episodes for the rest of LCA2 participants (7/10) occurred infrequently and were not reported during fMRI. Functional results are presented when the three individuals were/were not experiencing phosphenes to demonstrate the differences in human brain activity in the presence or absence of these internally generated light experiences.

As illustrated in Figures 3–5, all LCA2 subjects' responses to the checkerboard stimuli, showed an overwhelming amount of cortical activations covering the entire bilateral visual cortices in the presence of phosphenes (panels B and D). In contrast, there were limited cortical activations recorded in the absence of phosphenes (panels A and C). Comparison of the fMRI results for treated (panels C) to the untreated eyes (panels A) in the absence of phosphenes shows noticeably higher amount of significant activations for patients' treated eyes, similar to what we previously reported in other subjects (e.g. CH09, CH13, NP01, CH12) who do not regularly experience phosphenes [34], [37]. The one exception is NP02, who had received a low dose of the gene therapy vector (see Table 1) and whose treated eye activation may have been diminished by the macular hole, though visual function improvements after gene therapy were reported for this subject [32], [35]. fMRI results of all three of the unilaterally injected subjects when experiencing phosphenes clearly show that the magnitude and distribution of cortical activations were by far more extensive, intense and widespread throughout the visual cortices during phosphenes. Such episodes persisted regardless of the extent of retinal disease (including the macular hole in NP02) [32], [35], (Figure 3: Panels B and D). It is important to note that clinical fMRI results reported on CH08 [34] in a separate study involving unilateral administration, was carefully monitored to be free of phosphene episodes.

The cortical activation patterns produced while subjects were experiencing phosphenes are by far much larger and more extensively distributed in the brain than the activation pattern triggered by the same stimuli in normal sighted individuals. This is illustrated in Figure 6 where fMRI results from the left and right eyes of two normally sighted controls are presented. As expected, both normal subjects showed significant and symmetrically distributed bilateral activation. However, the amount and distribution of activation in the normal control subjects are noticeably less than what was observed in patients experiencing phosphenes. Also, as compared to sighted controls, the pattern of cortical activity for the LCA patients in the absence of phosphenes is drastically lower and limited more to the extra striatal areas.

As depicted in Table 1, phosphene experiences did not appear to be dependent on patients' age, gender, visual acuity, *RPE65* mutation type, concentration, or volume of vector they received for gene therapy. Although the frequency and characteristics of phosphene experiences were not queried before and after gene therapy, some LCA2 patients reported experiencing them “somewhat” less frequently.

In agreement with widespread cortical activation, all three patients reported interference with their vision while experiencing phosphenes. In fact, all patients stated that they have to wait for phosphene episodes to end before they can continue their activities. Earlier studies in patients with retinitis pigmentosa by Bittner et al [38], [39] also described that patients with frequent occurrence of photopsias or spontaneous phosphenes reported interference with their vision. The results of fMRI presented here (immense cortical activations) may explain the inabilities of subjects to see while experiencing phosphenes (saturated/paralyzed visual cortex). The pattern of cortical activations invoked by phosphenes along with patients' self reports on inability to see during phosphene episodes indicates that patients should be queried about their phosphene experience during clinical testing, as phosphenes could potentially interfere with results of standard measures of visual function (visual acuity, visual field testing). Such effects may explain the challenges of high test-to-test reproducibility in tests of Goldman visual fields in LCA2 patients described by Roman et al. [40]. Thus, similar to the recommendation previously set forth by Bittner et al. [38], [39], our fMRI results also strongly advocate the need for awareness of phosphene experiences in all patients with retinal disease.

Although the cause of phosphenes in LCA2 patients is unknown, we hypothesize that they are of retinal origin and due to their *RPE65*-related disease. One plausible explanation suggested by Bókkon [41] is that retinal phosphenes may be due to overproduction of the ultra weak spontaneous photons [42] that are constantly emitted from the free radical reactions of retinal cells without any external excitation. If these biophoton emissions exceed a certain threshold, it can appear as phosphenes to the subject.

In summary, phosphenes may occur in a significant proportion of LCA2 patients and the characteristics can take on several different forms and can have an impact on the patient's visual experience. Although we did not perform correlations between visual function and phosphenes, we hope data presented report show not only that phosphenes can impact fMRI results but they also can potentially affect other forms of visual function testing. Future gene therapy clinical trials for retinal degeneration should gather additional information on phosphene experiences and correlate this information with visual function test results. fMRI data also illustrate the potential negative impact that phosphene perception may have on vision testing. fMRI has proven to be a sensitive, non-invasive and reproducible test paradigm for these purposes and can complement standard visual function testing. In this study, fMRI confirmed the long-term effects of retinal gene augmentation therapy in two LCA2 patients who had received medium (CH08 & CH11) vector genome concentrations [35]. Ultimately, the incorporation of fMRI studies with standard retinal function test paradigms will reveal whether gene therapy or other interventions slow or halt the progression of disease and/or improve vision. Results from the present report, however, bring awareness to clinicians and researchers on the effect this phenomenon may have on patients' clinical assessment. Although it should be noted that, as reported by Bittner et al [38], phosphenes may be only one source of increased variability in vision testing and other factors such as patient or operator related variables could also lead to inconsistent test results.

Awareness of potential phosphene phenomena in patients with retinal diseases is particularly important with respect to evaluation of emerging therapies including gene, molecular, and cellular therapies.
